# High-*T*_c_ superconductivity in undoped ThFeAsN

**DOI:** 10.1038/s41467-017-00185-4

**Published:** 2017-07-31

**Authors:** T. Shiroka, T. Shang, C. Wang, G.-H. Cao, I. Eremin, H.-R. Ott, J. Mesot

**Affiliations:** 1 Laboratorium für Festkörperphysik, ETH Hönggerberg, Zürich, CH-8093 Switzerland; 2 Paul Scherrer Institut, Villigen, CH-5232 Switzerland; 3 Laboratory for Scientific Developments and Novel Materials, Paul Scherrer Institut, Villigen, CH-5232 Switzerland; 4 Swiss Light Source, Paul Scherrer Institut, Villigen, CH-5232 Switzerland; 5 Institute of Condensed Matter Physics, École Polytechnique Fédérale de Lausanne (EPFL), Lausanne, CH-1015 Switzerland; 6 Department of Physics, Shandong University of Technology, Zibo, 255049 China; 7 Department of Physics and State Key Lab of Silicon Materials, Zhejiang University, Hangzhou, 310027 China; 8 Collaborative Innovation Centre of Advanced Microstructures, Nanjing, 210093 China; 9 Institut für Theoretische Physik III, Ruhr-Universität Bochum, Bochum, D-44801 Germany; 10 Institute of Physics, Kazan Federal University, Kazan, 420008 Russia

## Abstract

Unlike the widely studied *Re*FeAsO series, the newly discovered iron-based superconductor ThFeAsN exhibits a remarkably high critical temperature of 30 K, without chemical doping or external pressure. Here we investigate in detail its magnetic and superconducting properties via muon-spin rotation/relaxation and nuclear magnetic resonance techniques and show that ThFeAsN exhibits strong magnetic fluctuations, suppressed below ~35 K, but no magnetic order. This contrasts strongly with the *Re*FeAsO series, where stoichiometric parent materials order antiferromagnetically and superconductivity appears only upon doping. The ThFeAsN case indicates that Fermi-surface modifications due to structural distortions and correlation effects are as important as doping in inducing superconductivity. The direct competition between antiferromagnetism and superconductivity, which in ThFeAsN (as in LiFeAs) occurs at already zero doping, may indicate a significant deviation of the *s*-wave superconducting gap in this compound from the standard *s* ^±^ scenario.

## Abstract (web summary)

Exploring the interplay between the superconducting gap and the antiferromagnetic phase in Fe-based superconductors remains an open issue. Here, the authors show that Fermi-surface modifications by means of structural distortions and correlation effects are as important as doping in inducing superconductivity in undoped ThFeAsN.

## Introduction

In the vast class of iron-based superconductors (IBS), only very few are superconductors in their original stoichiometric composition of compensated metals. Among them are LaFePO, with a critical temperature *T*_c_ ≃ 4 K^1^, LiFeAs, with *T*_c_ = 18 K^2^, and FeSe with *T*_c_ = 8 K^3^. Most of the other materials, including LaFeAsO, are antiferromagnets with *T*_N_ of the order of 100 K^4^. Given the itinerant character of charge carriers in IBS, superconductivity (SC) can be achieved via Fermi-surface tuning in two possible ways: through isovalent substitution of ions with different radii, or by injection of electrons or holes in the Fe planes of the magnetically ordered parent compounds^5–9^. In particular, for many 1111 materials, including LaFeAsO, enhanced critical temperatures are only achieved by F- or H-doping^5–11^. For most of the stoichiometric compensated-metal IBS, the superconducting properties deviate significantly from those where SC is induced by doping. For example, it has been claimed that the tendency towards antiferromagnetic (AF) order favors the so-called sign-changing (between electron and hole pockets) *s* ^±^-wave symmetry of the superconducting state, which originates from enhanced repulsive interactions between electron and hole bands^12, 13^. This interaction generally favors antiferromagnetism, but once disorder, pressure, or doping suppress the long-range AF order, the *s* ^±^-wave SC emerges. By contrast, for LaFePO, LiFeAs, FeSe and, as we show below also for ThFeAsN, where SC occurs in stoichiometric compounds without imposing pressure or doping, there are strong indications that, due to orbital effects, the superconducting-gap symmetry deviates significantly from the *s* ^±^ scenario^14–17^.

Very recently, the undoped compound ThFeAsN was found to exhibit an onset of SC at a remarkably high *T*_c_ of 30 K, as established by magnetic susceptibility and electrical resistivity measurements^18^. Considering that oxygen and selenium are typical ingredients of stoichiometric IBS materials, the absence of chalcogen elements in ThFeAsN is remarkable.

Since energy-dispersive X-ray (EDX) analyses of the synthesized polycrystalline TheFeAsN material indicate no distinct O-for-N substitutions, it is of obvious interest to establish why, despite the lack of formal or incidental doping, a fairly high *T*_c_ is achieved in this case and to what extent the magnetic correlations support, interfere, or compete with SC. Pure compounds such as ThFeAsN, for which reliable band-structure calculations are available, represent ideal candidates also for testing contending theories of IBS.

In the following, we report on the detailed microscopic investigation of ThFeAsN by employing muon-spin rotation/relaxation (*μ*SR) and nuclear magnetic resonance (NMR) techniques, which are used to probe the magnetic and electronic properties of ThFeAsN at a local level. As we show below, the experimental results indicate that in this material, strong spin fluctuations precede the onset of the superconducting phase and compete with it. The appearance of SC in ThFeAsN in its pristine form seems ultimately related to an appropriately tuned electronic band structure, which also clarifies the rare peculiarity of this system.

## Results

### Probing the intrinsic magnetism via zero-field *μ*SR

**Fig. 1:**
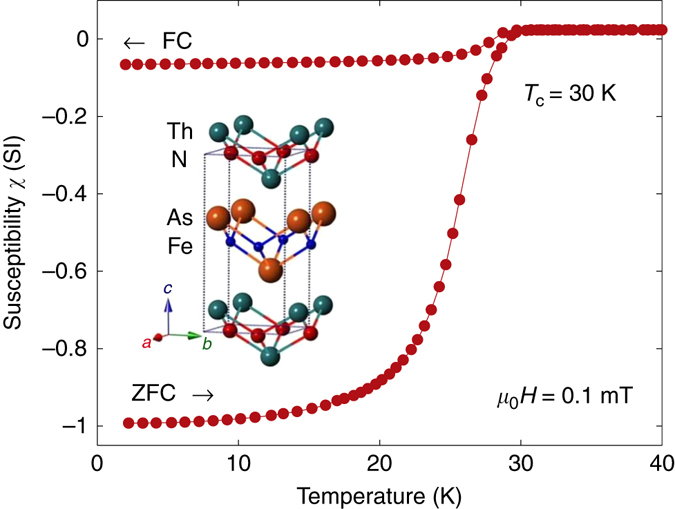
Magnetic susceptibility of ThFeAsN. Temperature dependence of the zero field-cooled (ZFC) and field-cooled (FC) dc susceptibility measured at *μ*_0_ *H* = 0.1 mT. The sample shows a sizable diamagnetic response and *T*_c_ = 30 K. Inset: structure of ThFeAsN showing the ThN and FeAs planes (adapted from Wang et al.^18^)

Preliminary susceptibility measurements *χ*(*T*) below 40 K were used to detect the onset of bulk SC. As shown in Fig. 1, both zero field-cooled (ZFC) and field-cooled (FC) data indicate a superconducting transition at *T*_c_ = 30±0.5 K, in good agreement with the originally reported value^18^.

**Fig. 2:**
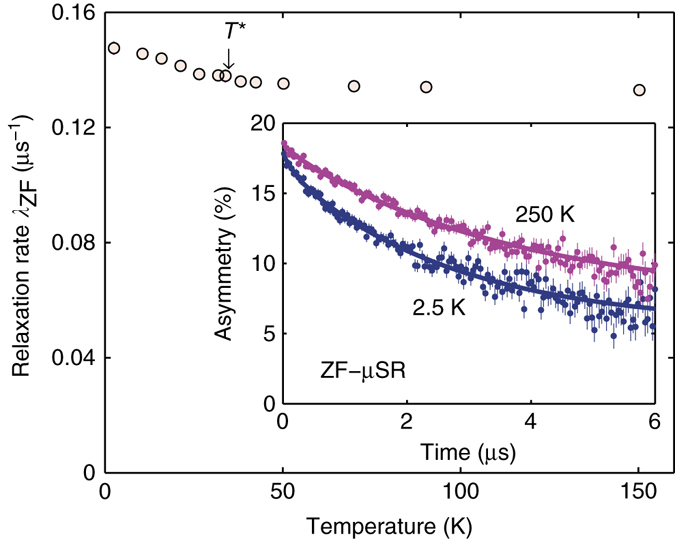
ZF-*μ*SR time spectra and relaxation rates. The zero-field relaxation rate *λ*_ZF_ is small and practically constant with temperature, with only a tiny increase below *T* ^*^. Inset: representative ZF-*μ*SR spectra at selected temperatures

By means of systematic zero-field (ZF)-*μ*SR measurements, sensitive to the material’s intrinsic magnetic properties, we could follow the evolution of a possible magnetically ordered phase. As shown in the inset of Fig. 2, the time-domain *μ*SR spectra generally exhibit exponential decays, which become more prominent as the temperature decreases. An ideal non-magnetic sample is expected to show a constant asymmetry. Real samples, however, invariably show small, mostly temperature-independent decays, attributed to nuclear magnetic moments or to tiny amounts of diluted ferromagnetic impurities, the latter commonly occurring in various IBS families^19, 20^. Their randomly oriented moments are known to create weak stray fields over the entire sample^21^, hence giving rise to muon-spin relaxation. In our case, magnetometry results are consistent with a 0.5% extra amount of Fe (i.e., below the detection threshold of powder X-ray diffraction (XRD) in the form of tiny dispersed clusters, which provide a temperature-independent average magnetic moment of ca. 0.03 *μ*_B_/Fe. Accordingly, our ZF asymmetry data were analyzed by considering the sum of two contributions: ${A_{{\rm{ZF}}}} = A_{{\rm{ZF}}}^{{\rm{sample}}} + A_{{\rm{ZF}}}^{{\rm{imp}}{\rm{.}}}$. The latter is relevant only at short times, but since its amplitude does not exceed 10% of the total signal, it is hardly discernible in the inset of Fig. 2. The ZF-*μ*SR signal of the sample is well described by exponential relaxations of the form (see inset in Fig. 2):

1 $${A_{{\rm{ZF}}}}(t){\rm{/}}{A_{{\rm{ZF}}}}(0) = {p_{{\rm{fast}}}}{{\rm e}^{ - {\Lambda _{{\rm{fast}}}}t}} + {p_{{\rm{slow}}}}{{\rm e}^{ - {\Lambda _{{\rm{slow}}}}t}},$$

where *p*_fast,slow_ and *Λ*_fast,slow_ are the relative weights and relaxation rates of muons implanted in two inequivalent sites, namely close to FeAs and to ThN planes, respectively^22, 23^. In agreement with these studies, we find *p*_slow_ ~ 15% at *T* = 5 K, decreasing with temperature.

The relaxation rate *λ*_ZF_ (≡ *Λ*_fast_), reflecting a possible magnetic order due to Fe^2+^ ions, is shown in Fig. 2. It is practically independent of temperature, exhibiting a marginal increase (of only ~0.02 μs^−1^) below T* = 35 K (see NMR results below for a definition of T*). Given the absence of applied magnetic fields in ZF-*μ*SR experiments, an increase in *λ*_ZF_ is usually attributed to the onset of AF order (local moment or spin-density wave). However, given its tiny value and the absence of coherent muon-spin precession below T*, it indicates a broad distribution of weak internal fields, i.e., no well-developed magnetic order in ThFeAsN. This is consistent with results of transport, magnetic^18^, and ^57^Fe Mössbauer spectroscopy^24^, studies, where no magnetic order was detected down to 2 K. Similarly to the superconducting F- or H-doped LaFeAsO^20, 25^, as well as to many other 1111 compounds^26, 27^, where a short-range magnetic order, vanishing with doping, is claimed to coexist with SC, also in ThFeAsN, the weak magnetism seems closely related to the onset of SC. Longitudinal-field *μ*SR experiments (not discussed here) indicate that in ThFeAsN too, the weak magnetic moments behave as static within the *μ*SR time scale. On the other hand, ThFeAsN clearly differs from pure LaFeAsO which, below *T*_N_, exhibits oscillating ZF-*μ*SR signals^25^, a signature of long-range magnetic order. From this comparison, it is evident that the undoped ThFeAsN already fulfills the conditions to sustain SC, which other 1111 compounds achieve only upon doping.

**Fig. 3:**
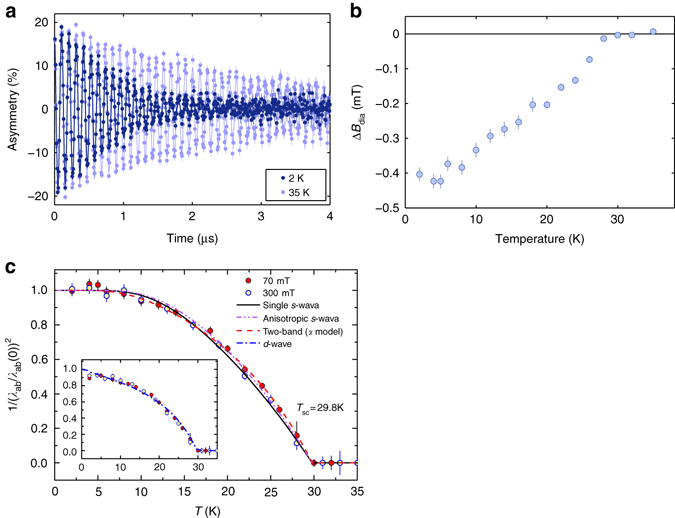
TF-*μ*SR spectra, diamagnetic shift, and relaxation rate. **a** Representative TF-*μ*SR spectra above and below *T*_c_ measured in 70 mT and relevant fits. **b** Diamagnetic field-shifts in the superconducting phase. **c** Temperature dependence of *λ* ^−2^, as calculated from the measured TF relaxation rate *σ*_sc_(*T*) at 70 mT (*red*) and 300 mT (*yellow*). *Lines* represent fits using a single-gap (*solid*) and two-gap or anisotropic *s*-wave model (*dashed*), the latter two showing a better $\chi _r^2$. The inset shows a fit using a *d*-wave model

### Probing SC via TF-*μ*SR

To explore the nature of SC in ThFeAsN, we performed a series of transverse-field (TF)-*μ*SR measurements from 1.6 to 35 K. A type-II superconductor exposed to an external magnetic field develops a regular flux-line lattice (FLL), which modulates the field distribution inside the material. Muons, which sample the FLL uniformly, experience an additional Gaussian relaxation *σ*_sc_, the latter being a measure of the absolute magnetic penetration depth *λ* and, hence, related to the superfluid density *ρ*_sc_ ∝ *λ* ^−2^ ^28, 29^.

The temperature dependence of *σ*_sc_ was studied in a 70 mT transverse field under field-cooling conditions, with additional data collected also at 300 mT. A preliminary field-dependence study of the *μ*SR depolarization rate at 2 K indicated that both fields are suitable for probing the intrinsic superconducting properties of ThFeAsN, since neither the decrease of the intervortex distance with field, nor the vortex-core effects^30^ are of significance below ~500 mT, considerably smaller than *B*_c2_ ~ 50 T (Cao, personal communication). Both data sets give comparable *σ*_sc_ values (see Fig. 3c) but, for a direct comparison with available data on other IBS compounds, we focus on the 70 mT case. Figure 3a shows two typical TF-*μ*SR spectra measured above and below *T*_c_, fitted by means of:

2 $${A_{{\rm{TF}}}} = {A_{{\rm{TF}}}}(0)\,{\rm{cos}}\left( {{\gamma _\mu }{B_\mu }t + \,\phi } \right){e^{ - {\lambda _{{\rm{ZF}}}}t}}{e^{ - {\sigma ^2}{t^2}/2}}.$$

Here *A*_TF_(0) is the initial asymmetry, *γ*_*μ*_ = 2*π* × 135.53 MHz/T is the muon gyromagnetic ratio, *B*_*μ*_ is the local field sensed by the implanted muons, *ϕ* is the initial phase, *λ*_ZF_ is a Lorentzian-, and *σ* a Gaussian-relaxation rate. As follows from the above ZF analyses (see Fig. 2), the Lorentzian relaxation reflects coexisting magnetic correlations and is significantly smaller than the SC-dominated Gaussian relaxation rate, *σ*. The latter comprises contributions from both the FLL (*σ*_sc_) and a small temperature-independent relaxation due to nuclear moments (*σ*_n_), determined above *T*_c_. Below *T*_c_, the FLL-related relaxation was obtained by subtracting the nuclear contribution from the Gaussian relaxation rate, i.e., $\sigma _{{\rm{sc}}}^{\rm{2}} = {\sigma ^{\rm{2}}} - \sigma _{\rm{n}}^{\rm{2}}$.

**Fig. 4:**
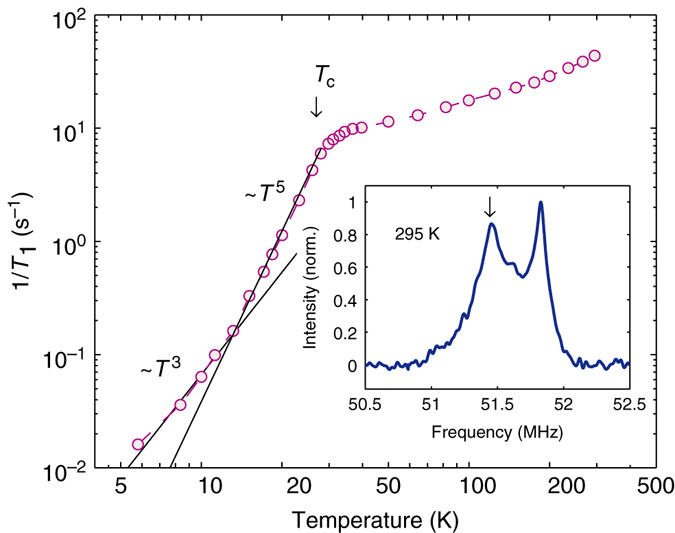
NMR relaxation rate and lineshape. Temperature dependence of 1/*T*_1_ relaxation rate measured at 7 T at the left peak of the ^75^As NMR lineshape (*arrow* in the inset). A similar 1/*T*_1_ behavior is found also for the right peak. The steep decrease of 1/*T*_1_ below *T*_c_ is compatible with a fully gapped superconductor. *Lines* indicate different power-law dependences, at different temperature regimes (see text for details)

Figure 3b depicts the diamagnetic shift below *T*_c_, whose value increases deep inside the superconducting phase. The simultaneous development of a flux-line lattice at low temperatures implies the appearance of *σ*_sc_, in turn reflecting an increase in 1/*λ* ^2^ (see Fig. 3c). For small applied fields (in comparison with *B*_c2_(0)) and hexagonal flux-line lattices, the two quantities are related by^29, 31^:

3 $$\frac{{\sigma _{{\rm{sc}}}^{\rm{2}}(T)}}{{\gamma _{\rm{\mu }}^{\rm{2}}}} = 0.00371\frac{{\phi _0^2}}{{\lambda _{{\rm{eff}}}^4(T)}},$$

with *λ*_eff_ the effective penetration depth. We recall that in anisotropic polycrystalline superconducting samples (as is the case of the layered ThFeAsN compound), the effective penetration depth *λ*_eff_ is determined mostly by the shortest penetration depth *λ*_ab_, the exact relation between the two being *λ*_eff_ = 3^1/4^ *λ*_ab_^32^.

Figure 3c shows the temperature dependence of the normalized superfluid density, ${\rho _{{\rm{sc}}}} \propto \lambda _{{\rm{ab}}}^{ - 2}$. The temperature-independent behavior of $\lambda _{{\rm{ab}}}^{ - 2}$ below 7 K indicates a fully gapped superconductor, thus excluding, e.g., a *d*-wave gap structure. Indeed, as shown in the inset of Fig. 3c, a *d*-wave model does not properly fit the experimental data, especially below 5 K. However, as can be seen in the main panel of Fig. 3c, single *s*-wave, two-gap *s*-wave or anisotropic *s*-wave gap models are all compatible with the observed $\lambda _{{\rm{ab}}}^{ - 2}(T)$ behavior. The better agreement of the two-gap model (or *s*-wave gap with substantial anisotropy) with the data is confirmed by the slightly positive curvature in *B*_c2_(*T*) close to *T*_c_, as derived from magnetization and electrical resistivity measurements, which also favor a multigap SC state. Given the two to five Fe-related bands crossing the Fermi surface, the occurrence of multiple SC gaps in iron-based superconductors is not surprising^9, 13^. Recent Fermi surface calculations^33, 34^ suggest a similar scenario also for ThFeAsN.

A comparison of different families of superconductors can be summarized in a so-called Uemura plot^35^. This type of representation tracks the dependence of *T*_c_ on the inverse square of the in-plane London penetration depth, $\lambda _{{\rm{ab}}}^{ - 2}$. Since $1{\rm{/}}\lambda _{{\rm{ab}}}^2(0) \sim {\rho _{{\rm{sc}}}}{\rm{/}}{m^ \star }$, with *ρ*_sc_ the superfluid density and ${m^ \star }$ the renormalized mass of the quasiparticles, a positive correlation between *T*_c_ and ${\rho _{{\rm{sc}}}}{\rm{/}}{m^ \star }$ was identified. In this diagram, ThFeAsN lies close to the data set for FeSe and, remarkably, also to the electron-doped LaFeAsO. This is not surprising given the almost identical results of the first-principle electronic-structure calculations for ThFeAsN and LaFeAsO^33, 34^. In view of this, the observation of SC in stoichiometric ThFeAsN without any additional doping is quite remarkable.

### Complementary findings from NMR investigations

As a complementary technique to *μ*SR, ^75^As NMR was used to locally probe the static (line widths and shifts) and the dynamic (spin-lattice relaxation) properties of ThFeAsN. The results of these measurements (at 7 T) confirm the features presented and discussed above, but provide also additional insight into the characteristics of SC of ThFeAsN.

Since ^75^As has a nuclear spin *I* = 3/2, the observed NMR line is broadened by a second-order quadrupole perturbation of the central Zeeman +1/2 to −1/2 transition, the satellites being much weaker and far apart. Both the two-peak lineshape (see inset in Fig. 4) and its variation with temperature are very similar to those of the ^75^As NMR lines observed in lightly F-doped LaFeAsO^36, 37^. Most importantly, consistent with the *μ*SR results, we observe only a marginal increase (<5%) in FWHM below 35 K, hence confirming the absence of magnetic order. The resonance frequencies can be modeled by *f* = *γH*_0_(1 + *K*) + *f*_Q_, with *K* the magnetic shift due to interactions between the probe nucleus and its electronic environment, and *f*_Q_ = −771 kHz, a temperature-independent second-order quadrupole shift.

**Fig. 5:**
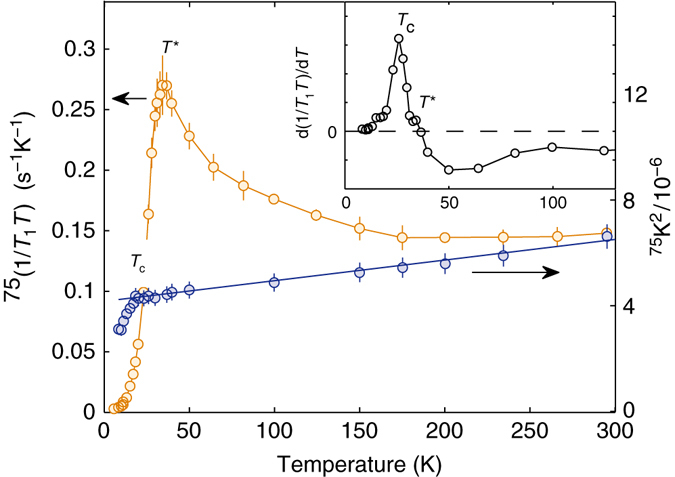
Different temperature behavior of 1/(*T*_1_ *T*) and *K* ^2^. Temperature dependence of 1/(*T*_1_ *T*) (*left* scale) and ^75^ *K* ^2^ shift (*right* scale) measured at 7 T. The peak in the former and the drop in the latter indicate *T* ^*^ and *T*_c_, respectively, which differ by ca. 5 K. The clearly different functional form of the two curves below ca. 150 K indicates the development of strong AF fluctuations. The strong low-temperature drop of 1/(*T*_1_ *T*) confirms the bulk character of SC, whereas the peak in the derivative (inset) indicates its sharp onset

Our most intriguing result is captured in Fig. 5, which compares the temperature dependencies of (*T*_1_ *T*)^−1^ and the squared magnetic shift *K* ^2^. Although according to standard theory, (*T*_1_ *T*)^−1^ ∝ *K* ^2^ ^38^, this is obviously not the case here. In the normal state, *K* ^2^ decreases weakly and linearly with decreasing temperature. (*T*_1_ *T*)^−1^(*T*), however, starts growing steadily below ~175 K (because of the chosen logarithmic scales in Fig. 4, this growth is less distinct, yet clearly recognizable also in $T_1^{ - 1}(T)$ data). This relative increase in relaxation is abruptly terminated at T* = 35 K, followed by a steep decrease, masking the onset of SC at *T*_c_(7 T) ≈ 27 K (identified by independent resistivity measurements). Note that a similar behavior of (*T*_1_ *T*)^−1^ was observed also in the lightly-doped LaFaAsO_1−*x*_F_*x*_^37^, or in the isoelectronically substituted LaFaAs_1−*x*_P_*x*_O class (T.S., manuscript in preparation). As argued in these cases, we also tentatively ascribe the extra relaxation to the growing influence of spin fluctuations. However, in the above examples, spin-fluctuations precede an incoming AF order, in turn a precursor of SC. By contrast, in ThFeAsN (where no AF order is detected), we postulate the opening of a gap in the corresponding spin-excitation spectrum at T*, exceeding *T*_c_, the latter clearly indicated by the sudden drop in *K* ^2^(*T*) at the onset of SC. These data suggest the onset of a superconducting state in competition with spin fluctuations.

Once the superconducting state is established below *T*_c_, a gradual decrease of *K* (see Fig. 5) hints at a spin-singlet pairing, for which the coupling between opposite spins implies a reduction of the local spin susceptibility. The temperature dependence of $T_1^{ - 1}$ (see Fig. 4) reflects a combination of growing gaps in the spectra of both spin and electronic excitations. Interesting is the transient *T* ^5^-regime for $T_1^{ - 1}(T)$, just below *T*_c_, where the spin gap is not yet fully developed.

## Discussion

One of the most intriguing questions regarding ThFeAsN is the occurrence of bulk SC in a stoichiometric compensated-metal compound without long-range magnetic order. Because of a strong nesting of the electron and hole bands (separated by the AF momentum), band-structure theory predicts that both undoped ThFeAsN and LaFeAsO should show a similar (*C*-type) stripe AF order^33, 34^. LaFeAsO indeed adopts a magnetically ordered ground state, while, as we have shown, ThFeAsN does not, but exhibits rather strong magnetic fluctuations. This key feature indicates a sizable renormalization of the electronic structure of ThFeAsN, as compared to that of LaFeAsO, beyond the density-functional theory. The simultaneous presence of fluctuations and the lack of magnetic order puts the stoichiometric compensated metal ThFeAsN into the same category as LiFeAs or FeSe, which also show strong correlations, although these correlations do not give rise to a magnetically ordered ground state, as instead is the case of LaFeAsO, CaFe_2_As_2_, etc. We recall also that in LiFeAs and FeSe the absence of magnetic order allows for an orbital-selective superconducting state to be realized, which involves a strong orbital renormalization and differentiation with respect to Cooper pairing on different orbitals. This contrasts with a conventional *s* ^±^ superconducting state, currently assumed for the F-doped LaFeAsO and K- or Co-doped BaFe_2_As_2_, where the gap is less orbital-dependent and simply changes sign between all-electron and all-hole pockets.

Clearly, the undoped ThFeAsN shares with optimally F-doped LaFeAsO, both the presence of magnetic fluctuations and the occurrence of SC. Yet, their similarity is only apparent. Indeed, while in F-doped LaFeAsO, the suppression of the original magnetic order is simply caused by doping, the mechanism of magnetic-order suppression in stoichiometric ThFeAsN has another, not yet known, origin. The missing information on the electronic renormalization in ThFeAsN can be provided by future ARPES or FT-STM measurements, once single crystals would be available. In any case, ThFeAsN currently offers the unique opportunity of studying the peculiarities of unconventional SC in the correlated IBS.

In conclusion, our results establish the proximity of SC with a competing state exhibiting sizable spin fluctuations in ThFeAsN. For many other iron-pnictide superconductors, it has been shown that magnetic order and SC coexist microscopically (see, e.g., refs. ^26, 27^). As outlined above, this is certainly not the case for ThFeAsN, whose lack of magnetic order places it in the same class with LaFePO, LiFeAs, and FeSe. Yet, as shown by NMR relaxation data, the proximity of a gapped spin-fluctuating phase to SC, does not exclude a spin-fluctuation meditated SC pairing in ThFeAsN. Microscopic *μ*SR and NMR measurements indicate also that the electronic excitation spectrum in the superconducting state is best described by a two-gap *s*-wave (or an anisotropic *s*-wave) model.

In a broader context, it was established that, e.g., in BaFe_2_As_2_, key structural features, such as the Fe-Fe distance and the As-Fe-As bond angle, vary in the same way under pressure or upon chemical doping, inducing similar electronic-structure evolutions in both cases^39^. While for most 1111 and 122 families the required electronic-structure modifications to sustain SC are achieved via chemical doping, for ThFeAsN, an appropriate combination of structural and electronic parameters results in an enhanced *T*_c_ already in its undoped state. This is not surprising, considering its *c*/*a* ratio of 2.11^18^, significantly reduced in comparison with other 1111 and 122 compounds, a difference ultimately due to the different ionic sizes of N^3−^ (1.46 Å) and O^2−^ (1.38 Å). The ThFeAsN case suggests that Fermi-surface modifications by structural distortions and correlationceffects are as important as charge doping in inducing SC in IBS compounds. Yet, the structural route to SC is so rare, because very few compounds do exhibit a suitable combination of structural parameters. Indeed, our recent high-pressure measurements have shown a sizable decrease of *T*_c_ with increasing pressure (Barbero. N. et al., manuscript in preparation), confirming that ThFeAsN has already the optimal parameters to achieve the highest *T*_c_.

## Methods

### Sample preparation

Polycrystalline ThFeAsN specimens with typical grain sizes 1–5 μm were synthesized by solid-state reaction methods, as recently described in ref. ^18^. The purity of precursors was checked via XRD, while the composition of the final product was determined via EDX spectroscopy. The room-temperature XRD of ThFeAsN reveals a tetragonal structure (*P*4/*nmm*) with *a* = 4.037 Å and *c* = 8.526 Å (see inset in Fig. 1) and no detectable impurity phases. The precise determination of the *N* content is rather challenging. While the possibility of N deficiencies cannot be absolutely ruled out, the fact that oxygen vacancies in our 1111 system are stabilized only under high-pressure synthesis suggests that N deficiencies, if present, are negligible.

### *μ*SR and NMR experiments

Experiments employing *μ*SR were done at the general purpose surface-muon instrument (GPS) spectrometer of the Paul Scherrer Institute (PSI) in Villigen, Switzerland. Once implanted in matter, spin-polarized muons (*μ* ^+^) act as microscopic probes of the local magnetic environment, which upon decay emit positrons preferentially along the muon-spin direction. The spatial anisotropy of the emitted positrons (i.e., the asymmetry signal) reveals the distribution of the local magnetic fields at the muon site^40, 41^. As for the NMR investigations, a broad-band spectrometer was used for the static (line widths and shifts) and the dynamic (spin-lattice relaxation) measurements. With a 100% isotopic abundance and a relatively large gyromagnetic ratio, the ^75^As nucleus (*I* = 3/2) was the probe of choice. In case of *μ*SR measurements, the error bars in the raw data were obtained from the counting statistics, while for the NMR from the noise levels and the frequency resolution. All the other error bars were calculated by using the standard methods of error propagation.

### Extracting superconducting parameters from *μ*SR data

The two-gap *s*-wave model provides *λ*_ab_(0) = 230(2) nm, with the two superconducting gap values being *Δ*_1_(0) = 3.4(2) meV and *Δ*_2_(0) = 6.5(3) meV, with weighting factors *w*_1_ = 0.40(2) and *w*_2_ = 0.60(3), respectively. From these, we find the following gap-to-*T*_c_ ratios: *Δ*_1_(0)/*k*_B_ *T*_c_ = 1.3 and *Δ*_2_(0)/*k*_B_ *T*_c_ = 2.5, with *k*_B_ = 8.62 × 10^−2^ meV K^−1^ the Boltzmann constant and *T*_c_ = 29.8 K. Similar values are obtained in the anisotropic single-gap *s*-wave case: *λ*_ab_(0) = 250(4) nm, *Δ*(0) = 4.8(5) meV, and *Δ*(0)/*k*_B_ *T*_c_ = 1.9. Since for an ideal BCS superconductor, the last ratio is expected to be 1.76, we conclude that ThFeAsN is a superconductor in the weak-coupling limit. The above values are in principle close to those of F-doped LaFeAsO, with *T*_c_ = 24 K and *Δ*(0) = 3.6 meV^42^.

From the results of magnetometry and *μ*SR measurements, other relevant SC parameters for ThFeAsN can be extracted. By using the formula *B*_c2_ = *ϕ*_0_/(2*πξ* ^2^), where *ϕ*_0_ = 2.07 × 10^−15^ Wb is the magnetic flux quantum, we estimate a superconducting coherence length *ξ*(0) = 2.57 nm. This rather small *ξ* value, combined with a large penetration depth *λ*_ab_(0) = 230(2) nm, as determined via TF-*μ*SR, indicate that ThFeAsN is an extreme type-II superconductor, with a Ginzburg-Landau parameter $\kappa = \lambda {\rm{/}}\xi \simeq 90$. By using this value for *κ* and the formula ${B_{{\rm{c}}1}}(0) = {\phi _0}{\rm{/}}\left( {4\pi \lambda _{{\rm{ab}}}^2} \right){\rm{ln}}\,\kappa$ ^43^, we also obtain the lower critical field *B*_c1_(0) = 13.4 mT, similar in magnitude to the *B*_c1_(0) values of other IBS compounds^8^.

### Data availability

The data that support the findings of this study are available from the corresponding authors upon reasonable request.
